# Challenges in delivering urban healthcare services during COVID-19 pandemic: a mixed-methods study in Ahmedabad, India

**DOI:** 10.1186/s12913-025-13147-0

**Published:** 2025-07-26

**Authors:** Sandul Yasobant, Krupali Patel, Ravina Tadvi, Hardi Thacker, Walter Bruchhausen, Deepak Saxena

**Affiliations:** 1 Department of Public Health Science, Indian Institute of Public Health Gandhinagar (IIPHG), Gujarat, India; 2 Centre for One Health Education, Research & Development (COHERD), Indian Institute of Public Health Gandhinagar (IIPHG), Opp. Air Force Head Quarters, Nr. Lekawada, Gandhinagar, Gujarat India; 3 Global Health, Institute for Hygiene & Public Health, University Hospital Bonn, Bonn, Germany; 4 Jindal School of Public Health and Human Development, O.P. Jindal Global University, Sonipat, Haryana India

**Keywords:** Community health workers, COVID-19, Community-based routine healthcare services, Urban health, Pandemic preparedness

## Abstract

**Background:**

The COVID-19 pandemic globally posed a great challenge to the existing healthcare system for delivering routine services. Community Healthcare Workers (CHWs) play an important role in delivering routine as well as COVID-19-related services at the forefront. Understanding the complexity of service delivery and the challenges faced by them is very crucial. This study aims to investigate the specific challenges for community-based routine healthcare services during COVID-19.

**Methods:**

The cross-sectional study was conducted in Ahmedabad City, Gujarat, from November 2021 to October 2022. A mixed-methods approach was adopted for carrying out this study. Ahmedabad was selected as the site of this study as it is a large populous city and has witnessed the outbreak of other diseases like CCHF and SARS, and it also experiences a significant impact of Covid-19.

**Results:**

A total of 150 CHWs were included in the study for the quantitative survey, and nine CHWs were interviewed to gather qualitative information. The results show the disruption in the community-based routine services, especially during the first wave. Increased workload (93% of CHWs) was a major challenge during COVID-19 was one of the biggest challenges from the provider side, while fear of leaving the house (97%) was reported as one of the biggest challenges from the demand side. During the first wave, services for Non-Communicable Diseases (NCD) were highly affected. CHWs reported that they were motivated to work in the pandemic to provide the necessary care and services to the community. Findings from this study also suggest the need for structured training in emergency preparedness, mental health support, and coordination skills, with policy implications for urban health systems' resilience.

**Conclusion:**

The results suggest that additional training for CHWs on pandemic response and infection control would be highly helpful during emergencies. Recruitment of trained human resources and intersectoral collaboration are very important for the appropriate management of and preparedness for such pandemics in future. These are some of the main pillars of strengthening the health system. This study uniquely contributes by documenting the use of CHWs in a large urban setting and highlighting innovative intersectoral collaboration mechanisms that emerged during the crisis. Further, this study provides an insight into the lessons learnt from the COVID-19 pandemic towards maintaining healthcare services during crises.

**Supplementary Information:**

The online version contains supplementary material available at 10.1186/s12913-025-13147-0.

## Introduction

The COVID-19 pandemic has challenged the existing healthcare system globally. It is crucial to ensure continuity of service delivery for routine and essential care to reduce excess morbidity and mortality during such a pandemic and preserve public confidence in the healthcare system [1]. Primary healthcare aims to provide comprehensive, accessible, community-based care that meets the health needs of individuals throughout their lives. Community-based healthcare services, often delivered by Community Healthcare Workers (CHWs), are a cornerstone of primary healthcare, especially in LMICs, by bridging the gap between communities and the formal health system. Healthcare workers, mainly CHWs, play a crucial role in the COVID-19 pandemic response, balancing the requirement for greater service delivery with preserving access to essential medical care and routine health services [2]. Increasing demand for healthcare services among COVID-19 patients disrupted the delivery of the routine services provided by frontline healthcare workers [3]. For instance, in India between February and April 2020, there was a dramatic drop in TB case detection by 69% and services related to non-communicable diseases [4], maternal and child health [5], and routine immunisation [6] were too grossly affected during the COVID-19 pandemic. During the conversion of hospitals (most private and public) to COVID-19 centers, community health workers (CHWs) were deployed for COVID-19, laboratories reduced testing of other diseases, and fear of getting infected arose among the community while seeking healthcare for conditions other than the COVID-19 infection. In addition, other areas like income, food security, social protection, and access to clean water and sanitation were also compromised [7].

The COVID-19 pandemic has brought to light flaws in the healthcare infrastructure, including a frequent shortage of healthcare workers [8], low community involvement, and a gap in leadership in the healthcare sector [9]. In Asian LMIC countries, it was challenging for individuals with comorbidities who required help from the intensive care unit to get the necessary treatment since ICUs had to meet the expanding demand [10]. Due to COVID-19, impediments to vaccine distribution and administration in LMICs have been highlighted as a lack of personal protective equipment among healthcare professionals, closed vaccine clinics, suspension of vaccination services, and shortages of healthcare workers occurred frequently [6]. For instance, measles cases have increased with COVID-19 in Cambodia since January 2022. According to the United Nations Population Fund (UNFPA), 1.4 million unwanted births occurred in 115 low- and middle-income countries because 12 million women worldwide could not get contraceptive services due to disruptions in access. There has been evidence of funds diverted from MNCH during earlier epidemics and outbreaks of infectious diseases, such as Ebola and Zika [11]. This redirection of funds has continued under COVID-19, further straining already fragile health systems [12].

Effective pandemic response necessitates multisectoral preparedness coordination, which involves diverse stakeholders deliberately collaborating to enhance health emergency preparedness by pooling their knowledge, expertise, and resources [11]. Multisectoral collaboration can be used to minimise the social and economic impact of a disruption [13]. By actively participating in multisectoral communication, collaboration, and research advancement, as well as advancing knowledge of the virus and disease, health systems can contribute to the response to an outbreak or a pandemic [13]. However, this collaboration emphasised the prevention of secondary transmission rather than the involvement of different actors. For instance, HIV/AIDS, avian influenza, and Ebola virus disease demonstrated that building strong health security requires close collaboration across sectors [11]. In addition to impacting the health system, the COVID-19 pandemic and related lockdowns affected agriculture, food security, transportation, and education as well. These sectors’ workforce was used to control coronavirus transmission; hence, a multidimensional effect required multisectoral collaboration [11]. Some countries were better prepared than others. In Indonesia, a presidential directive to strengthen the nation’s capabilities to prevent, detect, and respond to infectious diseases, pandemics, bioterrorism, and other chemical, biological, radiological, or nuclear dangers and threats had been signed by the President of the Republic already in June 2019 [11]. Furthermore, many countries have adopted telehealth to continue routine and essential health services [14]. In South Africa, restructuring the clinical flow was established to ensure the security of the patients.

While these global and regional experiences highlight systemic challenges, the Indian context presents a unique and complex case. India’s dual burden of communicable and non-communicable diseases, coupled with a resource-constrained public health infrastructure, made the delivery of routine healthcare services during the pandemic especially challenging. Despite these pressures, India’s primary healthcare reforms under the Ayushman Bharat initiative—particularly the establishment of Health and Wellness Centres (HWCs)—relied heavily on the mobilization and resilience of community health workers (CHWs) to sustain service delivery at the grassroots level [15]. However, there remains a paucity of documented evidence, particularly from large urban settings in India, on how community-based routine healthcare services were maintained and what contextual factors contributed to their continuity. Understanding these dynamics is essential, as India and other low- and middle-income countries (LMICs) continue to scale up primary health care (PHC) reform and move toward universal health coverage [16].

Thus, this study aims to investigate the challenges in delivering community-based routine healthcare services during COVID-19 in Ahmedabad city and show some factors of success in maintaining them. The findings are intended to inform strategies for strengthening urban primary healthcare systems and enhancing preparedness for future health emergencies, offering lessons of relevance for 2025 and beyond.

## Methods

### Research design

The cross-sectional mixed metho study involving both qualitative and quantitative approach was carried out to investigate the impact of COVID-19 on routine healthcare delivery in Ahmedabad City, Gujarat. The study was carried out from November 2021 to October 2022. Ahmedabad was selected as it is the eighth most populous city in India and the most populous in Gujarat, experienced a significant COVID-19 burden, has a complex urban health system and the city has faced some previous infectious disease outbreaks, providing a relevant context to study urban healthcare challenges.

### Study settings

The study was conducted in the Western Indian Ahmedabad city, Gujarat. Ahmedabad is the fifth most populous city in the country and the most populous city in Gujarat. Ahmedabad was the third fastest-growing city of the decade in 2010, as stated by the Forbes list. The whole city of Ahmedabad has been divided into six administrative divisions: Central, East, West, North, South and Northwest zone. Of the total six zones, two zones (i.e. west and south zones) were randomly selected to execute this research study.

Ahmedabad is managed by a corporate body called Ahmedabad Municipal Corporation (AMC). The health department of AMC is responsible for managing the city’s health system. The governmental human health system includes Urban Health Centres (UHC) and hospitals with medical colleges. Private hospitals and dispensaries are entitled to providing health care services along with the AMC.

### Study samples & sampling

#### Quantitative assessment

The survey was conducted at the community level among the CHWs. Multi-stage cluster sampling was carried out. First, the West and South zones of Ahmedabad were randomly selected. Within these two zones, a list of all UHCs was obtained, and a predetermined number of UHCs were randomly selected as clusters. From these selected UHCs, CHWs—including Accredited Social Health Activists (ASHA workers), Auxiliary Nurse Midwives (ANMs), Anganwadi Workers (AWWs), and Multi-Purpose Health Workers (MPHWs)—were randomly selected using lists provided by the UHCs. Efforts were made to ensure representation across various CHW cadres by setting minimum quotas for each cadre type within the total sample. However, their years of experience were not accounted for their recruitment in the study.

#### Qualitative assessment

For the qualitative component, In-depth Interviews (IDIs) were conducted. A purposive sampling strategy was used to select nine CHWs from the two selected zones who had also participated in the survey. Participants were selected to ensure representation of different types of CHWs (ANMs, MPHWs, ASHA worker) and varying levels of experience to capture a diverse range of perspectives.

##### Data collection

Data collection was done by trained field investigators with prior experience in public health research and qualitative interviewing.

#### Quantitative assessment

A structured questionnaire was used to collect the quantifiable information from the CHWs. The questionnaire, developed based on literature review and expert consultation, was pilot-tested with 15 CHWs (not included in the final study sample) in a non-selected zone of Ahmedabad to assess clarity, flow, and time taken, and minor revisions were made accordingly. A pilot testing of the tool was conducted in the vernacular language and based on the validated responses the final tool in the vernacular language was developed. To increase the robustness of the questionnaire the back translation were also conducted during the pilot phase. The questionnaire includes basic socio-demographic details, details of routine health services provided before the pandemic, services being provided during the different waves of the pandemic, demand side and supply side barriers in providing routine health care services, collaboration patterns and degrees of involvement of collaborators in the service delivery during COVID-19, and for quantifying the degree of task shifting, and motivation during pandemic activities.

#### Qualitative assessment

The IDIs were conducted using a semi-structured interview guide was prepared, pilot tested and then used for interviews. The semi-structured interview guide comprised basic details, routine responsibilities, functioning during the pandemic, human resources (HR), management measures for issues like shortage of drugs or manpower and intersectoral collaboration details. Interviews were conducted in a private setting convenient for the participants, mostly at their respective health centers, and lasted approximately 45–60 min. All interviews were audio-recorded with participant consent.

### Data analysis

#### Quantitative assessment

A descriptive analysis was conducted to find out about the services carried out during the pandemic and the demand and supply side barriers that arose. The analysis was carried out using STATA version 14.1.

#### Qualitative assessment

All the in-depth interviews were recorded, and transcription and translation into English were done for each interview. Thematic analysis was performed, and the data was coded based on that. Two independent researchers (KP & RT) coded the transcripts. Inter-coder reliability was established through consensus meetings to discuss coding discrepancies (if any), and a final coding framework was agreed upon with an independent third researcher (SY). A deductive approach was used to deduce the information regarding the roles and responsibilities and inter-sectoral collaborations emerging from the data.

Triangulation between quantitative and qualitative findings was used to enhance analytical validity.

### Ethical considerations

Ethical approval for this study was obtained from the Indian Institute of Public Health Gandhinagar - Institutional Ethics Committee (TRC/2021022/17). Permission was also obtained from the Ahmedabad Municipal Corporation. Written informed consent, explaining the study’s purpose, procedures, risks, benefits, and confidentiality measures, was obtained from each participant before data collection. Participant identifiers were masked during analysis and reporting to ensure confidentiality.

## Results

### Quantitative findings

A total of 150 CHWs were surveyed for this study, most of whom were ANM (45%), followed by ASHA (41%), MPHWs 7.3% and AWWs 6.7%. Most CHWs were females (93.33%) except MPHWs who are typically males, reflecting a gender distribution. in the Indian healthcare systems.

#### Supply-side challenges

The findings show that 52.5% of CHWs reported that the system-side challenges were a major reason for routine disruption during COVID-19. Increased workload during COVID-19 was one of the biggest challenges, as reported by 93% of CHWs. It was followed by the perceived fear (90%) of their families since they worried about the safety of CHWs, while among the CHWs themselves the fear of contracting the virus while delivering the services was at 74%. This indicates the need for health system lacuna in terms of safety policy for CHWs. The discomfort while wearing a face mask was reported as one of the challenges by 81% CHWs. The shortage of manpower and insufficient incentives for extra COVID-19-related work are reported in almost 70% and 60% of CHWs. Distances: The roads matter a lot to access health care facilities (HCFs), therefore, almost 62% of CHWs reported walking very long distances to provide the services at home/home visits and almost 56% of CHWs reported that patients have difficulty travelling to the facility. The factor perceived as a challenge by the lowest number of individuals was the lack of an Identity (ID) card (12%). The description of supply-side challenges as expressed by CHWs is shown in Table 1.

**Table 1:** Description of Supply-side challenges as expressed by CHWs of Ahmedabad, India during the COVID-19 pandemic (2021-22)

Supply-side Challenges	Sample	*n* (%)
Increased Workload	149	139 (93.29)
Family worried about their safety (CHW’s)	148	133 (89.86)
Facing discomfort in wearing a mask	148	120 (81.08)
Related CHWs deployed to provide COVID-19 relief	144	114 (79.17)
Fear of contracting virus while delivering services	149	110 (73.83)
Shortage of manpower	148	103 (69.59)
CHW has to walk long distances to provide THR/home visits.	105	65 (61.90)
Lack of incentives for CHWs in lieu of extra COVID-related work	141	83 (58.87)
Lack of co-operation from the beneficiaries	147	86 (58.50)
Difficulty in travelling to a facility	147	82 (55.78)
Lack of training for Village Health and Nutrition Day (VHND), home visits, or health promotion during COVID	139	60 (43.17)
Change in care-seeking policy for fever symptoms	142	55 (38.73)
Non-compliance with online technologies (Teleconsultation/Telemedicine)	122	39 (31.97)
Inadequate Personal Protective Equipment (PPE) for healthcare providers	147	42 (28.57)
Stock-outs of essential medicines or diagnostics	148	28 (18.92)
Lack of ID	131	16 (12.21)

Further, the perceptions regarding the challenges differ between the various groups of responders analyzed. The difference of opinion between ANM and ASHA was partly significant. Most ASHAs reported system-side challenges of non-compliance for online telemedicine and technology, significantly differing from the ANM’s report (*p* = 0.000 at 95% CI). Further, 58% of ASHAs and 42% of ANM reported walking long distances for home visits, a significant difference (*p* = 0.053 at 95% CI). These differences may reflect their distinct roles and field responsibilities.

#### Demand side challenges

Table 2 reflects that among the demand side challenges, defined as reported problems for maintaining routine services that arise from knowledge and attitudes, behaviour and circumstances in the target population for the services. Fear of leaving the house is one of the biggest challenges. Almost 75% of the CHWs reported that the beneficiaries were fearful of catching the virus, while 72% reported that community fear, mistrust and resulting refusal to seek care was a challenge. Language as a barrier on the demand side was only reported by 9% of CHWs.

**Table 2:** Description of demand side challenges as expressed by CHWs, Ahmedabad, India

Demand Side Challenges	Sample	*n* (%)
Fear of leaving the house	147	128 (87.07)
Fear of catching and spreading COVID-19, especially among families with infants	146	110 (75.34)
Community fear/mistrust/refusal in seeking health care	148	107 (72.30)
Reluctance to meet service providers	145	102 (70.34)
Financial difficulties during outbreak/lockdown	147	85 (57.82)
Limited use to technology/Internet access	133	50 (37.59)
Non-availability of service provider/CHW	131	34 (25.55)
Unavailability of services	140	33 (23.57)
Lack of Government-issued permit	137	23 (16.79)
Language Barrier	139	13 (9.35)

#### Routine RMNCH + N healthcare service delivery during the pandemic

The results show that most CHWs reported that Reproductive, Maternal, Newborn, Child and Adolescent Health (RMNCH + N) services were completely disrupted in the first wave. However, most CHWs opined them as partially disrupted in the second wave, compared to the first ^t^ wave. In contrast, most reported no disruption in service provision during the third wave.

Out of all the RMNCH + N services, institutional delivery was found to be the least affected service during the first wave. In contrast, Iron and Folic Acid (IFA) tablets, medicine and mask distribution were found to be as least disrupted service during the second wave. In contrast, family planning and contraception were found to be completely disrupted, opined by 75% of CHWs for the first wave, and 57% of CHWs felt partial disruption during the second wave. It was very interesting to see the results of the immunization services interruption. Nearly 82% of CHWs felt that immunization service was a completely disrupted service during the first wave, and the second wave received the highest partial disruption (45.8%) opinion. The results show that sick child services were affected during the first and second waves. Thus, routine RMNCH + N healthcare service delivery during the pandemic as reported by CHW is shown in Table 3.

**Table 3:** Routine RMNCH + N healthcare service delivery during the pandemic as expressed by CHWs, Ahmedabad, India

Specific service details	1 st Wave		2nd Wave			3rd wave	
	Completely Disrupted	Partially Disrupted	Completely Disrupted		Partially Disrupted	Completely Disrupted	Partially Disrupted
Family planning & contraception	75.2%	13%	31%		57%	3.4%	3.4%
Antenatal care	52.0%	19.5%	17.3%		43.3%	3.3%	4.1%
Postnatal care	55.3%	17.1%	18.1%		41.2%	0.8%	1.6%
Facility based births	43.3%	7.2%	13.1%		31.3%	4.0%	1.0%
Routine immunization services	81.7%	8.3%	20.2%		45.8%	4.2%	1.7%
Identification & registering new pregnancies, births & deaths	51.3%	11.8%	8.9%		36.6%	2.5%	3.4%
Sick child services	66.9%	17.4%	14.9%		55.3%	1.8%	3.6%
Management of moderate and severe malnutrition (THR distribution)	79.6%	5.4%	39.3%		33.7%	6.1%	5.1%
IFA tablet distribution, medicine or mask distribution.	42.9%	13.4%	13.2%		28.1%	3.4%	3.3%

#### Interruption in services for Communicable & Non-Communicable (CDNCD) diseases

During the first wave, NCD services were highly affected as most (80.9%) of CHWs reported that NCD diagnostics and treatment were disrupted. Further, 80.9% of CHWs reported that outbreak detection and control of non-COVID diseases was completely disrupted. Interestingly in the first wave, complete disruption of tuberculosis case detection and treatment was reported by 64% of CHWs, however, with this figure it was the least disrupted. The second least interrupted service in the first wave was– besides planned indoor residual spraying (IRS) for malaria control (70.4% disruption) the mobilization of the community for accessing healthcare-related treatment and services in health centres (71.9% disruption). A similar same pattern was found in the second wave. However, community mobilization & facilitation of healthcare service access in health centers was found to be with the highest disruption in the third wave. So it seemed to be most difficult to take up again services that highly depend on community cooperation. Table 4 shows the routine CDNCD health care service delivery during the pandemic as expressed by CHWs.

**Table 4:** Routine CDNCD healthcare service delivery during the pandemic CHWs, Ahmedabad

Specific service details	First Wave		2nd Wave		3rd wave	
	**Completely Disrupted**	**Partially Disrupted**	**Completely Disrupted**	**Partially Disrupted**	**Completely Disrupted**	**Partially Disrupted**
Outbreak detection and control (non-COVID diseases)	80.9%	3.5%	24.4%	44.5%	4.2%	4.2%
Continuation of established ARV treatment	77.7%	8.2%	21.5%	53.2%	3.5%	4.7%
TB case detection and treatment	64.1%	12.5%	17.2%	48.4%	3.3%	2.5%
Malaria diagnosis and treatment	75.4%	8.2%	21.4%	49.2%	4.2%	2.5%
Implementation of planned campaigns for distribution of insecticide treated nets	75.8%	9.2%	21.3%	48.4%	4.2%	3.3%
Implementation of planned campaigns for indoor residual spraying (IRS)	70.4%	12.3%	22.0%	47.5%	6.9%	4.3%
Implementation of seasonal malaria chemoprevention campaigns (SMC)	73.2%	14.8%	26.4%	49.1%	3.7%	6.5%
NCD diagnosis and treatment (e.g., hypertension, diabetes, asthma, chronic obstructive pulmonary disease, coronary artery disease)	80.9%	9.9%	25.8%	47.6%	2.5%	6.6%
Mobilization of community & facilitate them in accessing health related services available at health centers.	71.9%	7%	28.5%	39.7%	10.4%	8.7%

##### Duties in COVID-19 surveys and management of essential services

Out of 150 CHWs, 98 (76%) of CHWs reported that they were involved in survey activities related to COVID-19. Almost 77% (*n* = 94) were doing line listing of COVID-19 patients and 76% were taking migration details. Further, 78% of the CHWs reported that they were involved in COVID-19 immunization. Almost 87% of the CHWs said their routine work was affected due to their involvement in activities related to COVID-19. Moreover, on inquiring with the CHWs who received the support, they shared they mainly received manpower as additional support. Very few reported that they received transportation and food. Further, they were also inquired on how they managed the essential services in the absence of additional or limited support, the majority of CHWs reported that they did overtime and mainly provided on-call services.

#### Communication and information

As shown in the Table 5 below, the main communication mechanism was WhatsApp. Almost 71% of CHWs reported receiving information from their supervisor via WhatsApp followed by in-person meetings (63%). Although the government provides calling services, only 41% of CHWs reported their supervisor calling them to provide the information.

**Table 5:** Information about communication methods as expressed by CHWs, Ahmedabad, India

Information received from	*N*	Frequency	%
In-person meetings with superior	128	81	63.28
WhatsApp group with superiors	128	91	71.09
Call by superior	128	52	40.63
A video conference app with superior (G-meet, Webex)	128	5	3.91
Mass media (newspapers/radio/TV)	127	16	12.60

#### Motivation to work during the pandemic

The issues like lack of substantial remunerations, delay in supply of protective equipment and fear of infection was there yet community health workers were keen on delivering the services majorly due to the intent to work for community or ‘community service’. Almost 67.5% of CHWs stated that they were motivated to work in the pandemic as they wanted to provide the care and services to the community. Much lower response rates had other options since26% of CHWs worked due to ethical obligation and 25% worked due to their own positive attitude. Only 11% of CHWs reported that incentives motivated them to work and 19.5% reported that supervisors motivated them to work during the pandemic. The reasons for motivation to work as reported by the CHWs is shown in Table 6.

**Table 6:** Reasons for motivation to work for CHWs during the pandemic, Ahmedabad

Motivation to work during a pandemic	*N*	Frequency	%
Community services	126	85	67.46
Supervisors motivated them	128	25	19.53
Family support	128	22	17.19
Ethical Obligations	128	33	25.78
Incentives	127	14	11.02
Own positive attitude	126	32	25.40

When asked what they suggest to manage future pandemics, the majority responded that incentives will be the best motivation to work. Moreover, proper salary, permanent job, security of staff, training and transportation would be even more appreciated. This is also aligned with supply-side challenges they mentioned where they mainly shared their concerns over fear and health security.

#### Intersectoral collaboration

As shown in Tables 7 and 103 (almost 89%) of CHWs shared that they collaborated with teachers during COVID-19. The collaboration with police was reported by 61 (71%) CHWsand with NGO 19 (25%). The type of collaboration with these other actors ranges from not linked to fully linked. Thirty per cent of health staff reported that there were fully linked with teachers and not only working together, but also preparing the plan mutually and sharing human resources as well. Only 4% of CHWs reported that they were not linked with teachers. Almost 42% of CHWs menioned that they collaborated with teachers during COVID19 and worked together as a formal team with specific responsibilities. In the case of collaboration level with police, 64% reported that they were working informally with police to achieve their common goals, whereas the only 2% reported there were not linked with police at all. Only 19 CHWsreported that they did some kind of collaboration with NGOs, out of which 32% reported that they collaborated to work together as a formal team with specific responsibilities. Almost 16% said they didn’t link with any NGO at all, whereas 16% said they were fully linked with NGOs to work together with mutual planning and shared staff.

**Table 7:** Level of collaboration of CHWs with stakeholders

Level of Collaboration	With teachers (*N* = 103)	With police (*N* = 61)	With NGO (*N* = 19)
Not linked	3.90	1.70	15.80
Communication (Share information only)	6.80	9.80	26.30
Cooperation (work together informally to achieve common goals)	17.50	63.90	10.50
Collaboration (Work together as a formal team with specific responsibilities)	41.80	16.40	31.58
Fully linked (Work together as a formal team, mutually planning and sharing staff)	30.10	8.20	15.79

Further, the frequency of meetings with collaborating actors was inquired additionally. As shown in Table 8, the results show that CHWscollaborating daily with teachers, police and NGOs were reported to be 82%, 61% and 42%, respectively. The frequency of collaboration with teachers and police was found to be very low, whereas that with NGOs was found at 16%.

**Table 8:** Frequency of collaboration among stakeholders as expressed by CHWs, Ahmedabad

Frequency of collaboration	With teacher (*N* = 103)	With Police (*N* = 61)	With NGO (*N* = 19)
No contact	5.88	3.23	15.79
Daily	82.35	61.29	42.11
Twice weekly	6.86	17.74	15.79
Once weekly	2.94	3.23	15.79
Twice monthly	1.96	12.90	0
Monthly	0	1.61	10.53
Once monthly	0	0	0

### Qualitative findings

A total of 9 CHWs were interviewed. Out of nine, five were ANMs, three were MPHWs and one was ASHA. The work experience of ANM was 4–7 years and 9–13 years of MPHW. Based on the Key Informant Interviews’ responses, three themes were derived: (1) Impact of COVID-19 on their work and how they were managing, (2) Their work responsibilities in COVDI-19, and (3) Management support for COVID-19.

#### Impact of COVID-19 on regular activities of CHW and coping mechanism

CHWs universally reported that their routine activities, such as OPD services, Mamta Diwas (Village Health and Nutrition Day - VHND), Antenatal Care (ANC) and Postnatal Care (PNC) visits, were severely affected, especially during the first and second waves. This aligns with the quantitative data showing high service disruption.

*“In the first and second wave*,* our work was affected but from the third wave it started gradually as normal routine” [AN3]*.

CHWs described adapting their methods to maintain essential MCH services. Telephone consultations and socially distanced home visits became common, underscoring the limited telehealth adoption reported quantitatively (mostly phone use). The working of the staff from Anganwadi centres points t intra-sectoral collaboration at the ground level.

*“We were going to Anganwadi and to society to do ANC & PNC visits. ASHA workers were informing them the prior day. We were maintaining social distance and wearing PPE while checking them”. [AN4]*

When asked about their workload during COVID-19, most of them expressed that it increased significantly, specifically in the second wave. Even their working hours were extended, and they had to return on call. ASHA workers also shared that they had to report on how many of the COVID-19 positive patients were in-migrants and out-migrants in their area in addition to the COVID-19 survey in the second wave. They were also asked to survey the COVID-19 vaccination status in their area. In addition, they had to search for and bring the unvaccinated population to the centre for vaccination. This illustrates the immense pressure and diversification of tasks.

*“During the second wave*,* CHWs undertook multiple concurrent responsibilities*,* including contact tracing*,* vaccination*,* home visits*,* and conducting COVID-19 surveys. They also monitored population movement within their areas*,* tracked infections*,* recorded vaccination status—including first and second doses—and facilitated home-based vaccinations for individuals who did not attend scheduled sessions.” [A1]*

#### Work responsibilities for CHWs in COVID-19

CHWs said that they were usually conducting a survey on COVID-19 positive patients, going for vaccination and helping in testing mainly. They also mentioned providing the patients with kit which mainly included medications for fever and other drugs prescribed for the covid patient. Some CHWs also described taking on informal roles, such as monitoring quarantined individuals and even delivering essential supplies, reflecting a deep commitment but also a blurring of roles due to systemic pressures.

“*We were doing a survey among passengers who were coming from outside of the city or country. Also had to provide the information to them for 14 days of quarantine. And if they became positive then they had to visit them. Also had to give them hukam namu or ‘ultimatum’ to not go out for 14 days. Then I had to go to CHC for testing work. Also were preparing the RTPCR report”. [M1]*

*“We were referring the patients*,* prioritizing oxygen demand*,* providing kit to COVID-19 positive patients. Also providing sanitizer*,* gloves*,* mask etc. to COVID-19 and their contact person. We had to do additional work of COVID-19. We were taking the history of immigrants from another state or country. Also had to go to houses to stick the stickers at positive households”. [AN4]*

Beyond their formal job roles, the CHWs also had to play some informal roles, for example, keeping watch on positive patients. A few of them also shared that they did the job of security guard during the first wave as the awareness of the disease spread and infection was still very limited. One ASHA worker, one ANM and two MPHWs exprlained that they even did the job of food delivery and courier for patients in the first wave, so that those did not go out from the house. Most of them were also assigned the duty of COVID-19 testing.

*“We were also asked to do follow up of COVID-19 patients. Sometimes*,* it happened that patients were not listening and were going out of the house. In such a situation*,* we had to be there to perform guard duty. In the initial stage when COVID19 was not prevalent much at that time we were arranging chair and seating near to their house (liken in their society) during afternoons without their knowledge”. [AN4]*

According to ANM, they have contact details of the suspected populations to reduce unnecessary contact and fasten the survey. For this, the ANM were first calling and taking some details on the phone and then visiting these persons for details and sending them for testing if required. The posting was done in different areas for testing and survey. Public places such as railway stations or bus stations were targeted for mass screening. Few of the ANM were posted there for testing. One of them said sometimes it was very tiring to go from one place to another in the absence of a vehicle.

*“We were posted different places when testing started. Either we were on bus stand or railway station or crossing or bus stop”. [AN1]*

Since vaccination is one of the main interventions to control the surge of COVID19 all the CHWs were engaged in the vaccination task actively. Besides this, active searching of non-vaccinated parts of the population was performed by these CHWs.

*“We were also going in vaccination. First it started in our center*,* then in schools*,* then it started in Anganwadi”. [AN5]*

*“How many people vaccinated? Who took the second dose and who didn’t? If someone doesn’t come for vaccination*,* we were usually going to their home and bringing them for vaccination”. [A1]*

#### Management support for COVID-19

Despite limited financial incentives, CHWs reported being driven by self-motivation and a sense of duty. This corroborates survey finds where 67.5% cited “community service” as their primary motivator. The findings suggest the level of self-motivation and dedication for the community service among the CHWs.

*“My family members… were worried… But for me my duty is most superior. I am highly motivated to serve society… Serving people is my religion.” [A1]*.

*“Self-motivation*,* however*,* it would be good if they can just give some award or appreciation for our work during COVID-19.”. [AN1]*

The finding that 43% of CHWs reported a lack of training for managing routine services during COVID-19 was strongly corroborated qualitatively. Most CHWs stated they received no formal, planned training for pandemic response, relying instead on informal instructions from medical officers or guidelines shared via WhatsApp.

*“*CHWs commonly reported that they did not receive formal training for pandemic preparedness. Informal instructions through WhatsApp or verbal briefings from medical officers served as the primary means of guidance.*”*

The lack of systematic training highlights a key gap in preparedness and underlines the need for stronger institutional support in future health emergencies.

Regarding additional staff, CHWs confirmed receiving support from other FHWs, MPHWs, and Sanitary Inspectors. The extensive intersectoral collaboration reported quantitatively with teachers and police was vividly described. Teachers assisted with surveys, and police provided support during public interactions and vaccination drives, especially in uncooperative areas.

- An ASHA worker noted: *“Teachers were also coming… We were also going for the survey together house to house…”* [A1].
- An ANM detailed police involvement: *“Police were also supporting*,* but only for vaccination… in the slum area people were not that much cooperative so we had to call police…”* [AN3].

Non-Governmental Organizations (NGOs) were also mentioned as playing a key role in supporting survey efforts and helping to convince hesitant patients. The quantitative data reinforce this context, with high levels of fear among CHW, such as fear of leaving the house (87.07%), fear of infection (75.34%), and community mistrust (72.30%) which further created a difficult environment for service delivery. CHWs were often required to engage with reluctant individuals (70.34%) and address widespread hesitation, likely adding to their emotional burden. These challenges, at times mitigated with the support of NGOs, highlight the indirect yet significant psychological impact on CHWs also reported during qualitative survey.

- One ANM shared her fear: *“I felt depression as my daughter was only three years old. I was scared that because of me*,* what if she would be infected.”* [AN1].This fear for family safety was a strong quantitative finding as well (90%).

Suggestions for future pandemic preparedness centered on adequate and trained human resources, timely material supply (PPE, vaccines), logistical support (transport, food, water), better communication strategies with the public, and, importantly, improved staff remuneration and security.

- An ASHA worker emphasized: *“Additional staff is main requisite… staff should be trained on how to communicate… Stock of vaccination is very crucial…”* [A4].

## Discussion

This mixed-methods study provides crucial insights into the challenges faced by CHWs in delivering community-based routine healthcare services during the COVID-19 pandemic in urban Ahmedabad, India. It highlights the significant disruptions, the resilience and motivation of CHWs, the critical role of intersectoral collaboration, and key areas for strengthening health system preparedness for future emergencies. The findings hold relevance yet on date and beyond, as lessons learned from the pandemic continue to shape strategies for robust primary healthcare in LMICs.

### Service disruptions and CHW workload

A significant finding was the disruption of routine services in the three waves of the COVID-19 pandemic. Outbreak detection of non-covid diseases such as TB, Malaria, HIV/AIDS, and NCDs was highly disrupted (80.9%) during the first wave. The primary reason was again the deployment of CHWs for COVID-19 relief and the lack of staff which can manage these services. Bisht et al. and Babalola et al. [17, 18] highlighted that the possible cause for low testing on TB was that most laboratories prioritized COVID-19 testing. During the first wave, the services related to RMNCHA + N, such as family planning and contraception, routine vaccination, and Take-home Ration (THR) distribution, were severely impacted; however, other services associated with ANC, PNC, facility-based births, IFA tablet distribution, and sick child services were least impacted. This could be due to cooperation of Angan wadi staff as mentioned by CHWs in their interview. The usage of tele-health may have contributed to the minimal disruption in the counselling noted by CHWs. The gradual resumption of services by the third wave indicates some level of system adaptation, though challenges in re-engaging communities for services like mobilization persisted. The increase in the CHW workload due to COVID-19 duties, coupled with existing manpower shortages, was a primary challenge, consistent with Kundapur et al. 2022 [19]. Qualitative findings vividly portrayed CHWs juggling routine tasks, extensive COVID-19 surveys, contact tracing, and vaccination drives, often with extended hours and insufficient support. This underscores the need for clear protocols on surge capacity and workload management for CHWs during health crises.

### CHW motivation, psychological Well-being, and support

Despite considerable risks and challenges, CHWs exhibited strong motivation, predominantly driven by altruism and a sense of duty to the community (67.5%). This intrinsic motivation is a vital asset but should not be exploited. The finding that families were highly concerned for CHWs’ safety (90%) and that CHWs themselves feared contracting the virus (74%) highlights the significant psychological burden. This fear of transmitting the virus to families has been similarly documented among frontline health workers in Bangladesh by Tune et al. (2022) [20], emphasizing the interconnectedness of personal and familial concerns with work motivation and mental health during pandemics. The qualitative data, revealing instances of depression and anxiety, further underscores this. These findings align with studies like Dhillon PK, et al. (2024) [21] on the psychological resilience of frontline healthcare workers in India, indicating a need for structured mental health support systems. While our study did not systematically measure job satisfaction, the CHWs’ suggestions for better incentives, job security, and recognition are critical inputs for improving it, a factor explored by Revadi G, et al. (2024) in their tool development for CHW job satisfaction assessment [22].

### The crucial gap: training and preparedness

On the provider’s side, a significant finding was that no formal training had been given to CHWs regarding the pandemic. The confidence in being well prepared is also an important non-financial incentive. In our study, the system issue of not providing appropriate training to manage the pandemic situation was reported. The mentioned Haryana study had found that training is an important external motivation factor. Our results indicate the importance of training and preparedness in emergencies because 43.17% said they had not received any training for organizing or managing VHNDs or Home Visits. This is a major system-level failure. While CHWs adapted, relying on informal guidance, the absence of structured training likely increased stress, compromised efficiency, and potentially impacted service quality and safety. Effective preparedness requires proactive investment in training CHWs on infection prevention and control (IPC), risk communication, use of PPE, managing new tasks, and adapting routine service delivery protocols. The confidence derived from being well-prepared through such training can also act as a significant non-financial incentive, fostering a sense of competence and reducing anxiety.

### Intersectoral collaboration: a key success factor

Further, we have measured the level of collaboration for the control and prevention of COVID-19 between sectors such as the education department, police department and NGOs in the three pandemic waves. Another mechanism to manage the pandemic-related intersectoral collaboration functioned between CHWs and non-healthcare workers. Usually, in India, all government teachers must participate in a census survey. That is why they were chosen as potential supporting human resources for COVID-19 surveillance. Such support was not observed in any other report or study so far. The police department is usually part of the outbreak investigation team, so collaboration with police officers was established during COVID-19. Police officers were the first responders carrying out government directives to contain the spread of the disease, including lockdown orders. The systematic review conducted by Laufs & Waseem, 2020 as well recommended that the police department should collaborate with the public health sector for better preparedness and management of public health emergencies such as COVID-19 [23]. NGO workers collaborated with police and teachers to convince the people to follow COVID-19 appropriate behaviours and guidelines. Food distribution, fund allocation, and staff training were provided by NGOs [19, 24]. A study in Ethiopia also mentioned NGO and other sectors’ involvement in the fight against COVID-19 [25]. Another study from India too showed that states had an ‘COVID-19 Army’ or a designated group was set up to handle contact tracing, data analysis, and other tasks with the assistance of educators, the tax department, police, NGOs, and political organizations [19].

### Telehealth: limited adoption and untapped potential

In line with previous studies, our data from the present study also demonstrated that the primary reason for the collapse of routine services was regular staff from other health services deployed to provide COVID-19 relief [17, 26]. However, during an in-depth interview, we confirmed that services related to ANC and PNC were managed via telephone. This suggests that COVID-19 pandemic increased the use of tele-health [8, 19]. The quantitative data showed non-compliance with online technologies/telemedicine as a challenge reported by nearly a third of CHWs, particularly ASHAs. This divergence may stem from the cadre-specific roles and responsibilities where ASHAs, being community-based and engaged in household-level outreach, often face greater challenges in accessing and using digital tools compared to ANMs, who are facility-based and focused on clinical care. This suggests that while basic mobile communication was used, more advanced telehealth solutions faced barriers, possibly due to a lack of CHW training, device access, digital literacy among CHWs or the community, or connectivity issues. These role-specific and technological barriers directly impact service delivery, such as delays in case reporting and disease surveillance.

### Complementarity of quantitative and qualitative findings

Furthermore, underuse of telemedicine also risks missed follow-ups for chronic diseases and maternal health. These findings align with previous research indicating that ASHAs bear a disproportionate burden in last-mile [27–29] service delivery, especially under strained capacity during health emergencies. Expanding telehealth capacity among CHWs requires addressing these barriers through training, technological support, and ensuring equitable community access.

The mixed-methods approach provided a comprehensive understanding. Quantitative data highlighted the extent of service disruptions, challenges, and collaboration patterns. Qualitative findings offered depth, explaining *how* CHWs coped (e.g., adaptive field strategies for MCH), the *nature* of their increased workload, the *reasons* behind their motivation despite minimal incentives, and the *operational realities* of inter-sectoral collaboration and lack of training. For example, while quantitative data showed 79% CHWs deployed for COVID relief, qualitative data detailed the specific tasks (surveys, contact tracing, etc.) and their impact on routine work. Similarly, the high fear levels reported quantitatively were contextualized by personal stories of anxiety in the qualitative interviews. This synergy between methods strengthens the validity and richness of the findings.

### Novelty and relevance for future preparedness

Documenting specific adaptations and challenges in maintaining routine services across different pandemic waves. These findings are not merely descriptive of a past event but offer actionable insights for strengthening primary healthcare systems to be more resilient against future shocks. The challenges faced by CHWs– workload, safety, training– and the enabling factors like collaboration are timeless issues in public health emergencies.

### Implications for primary healthcare reforms and policy

The findings have significant implications for ongoing PHC reforms in India (e.g., Ayushman Bharat, HWCs) and other LMICs. These reforms rely heavily on CHWs like strengthening of the CHW cadre by training and capacity building, integrating the preparedness at the primary healthcare level, preparing frameworks for intersectoral collaborations that needs to be in place at the local level, and integrating the technology in scope of work of work of CHWs.

### Limitations of the study

The findings are from 2 zones so the results cannot be generalised. The data collection was carried out in mid- 2022, and the 1 st and 2nd wave of pandemic happened between early 2020 and 2021 so the participants might have recall bias. The psychological impact of the CHWs was not assesses during the study using validated tools.

## Conclusion and recommendations

This study analysed the causes and successful countermeasures for the disruption of routine health services at community level health services during the first wave of COVID-19 which had resumed fully by the end of the third wave. The disruption varied between services and was lowest in perinatal care, highest in family planning and TB detection, mainly due to the use of human and lab resources for COVID-19. Service disruption was highest in the first wave, but gradual redemption was seen during the 3rd wave. The results suggest that training for CHWs during emergencies, recruitment of further trained human resources and inter-sectoral collaboration are very important for appropriate management and preparedness of such pandemics in future. These are the crucial pillars of strengthening the health system for such emergencies. The study especially highlighted the importance of collaboration between health and non-health sectors and actors, as support from teachers played a vital role in relieving the overburdened CHWs at the grass-root level, specifically for the survey and tracing cases. At the same time, police officers helped CHWs and teachers in case of discordance in public. NGOs in and outside health provided essential services such as food supply. CHWs are vital and corner stone for building the health system’s resilience. Although this study highlighted their self-motivation, it’s an open call for appropriate authorities to implement structured capacity building programs on pandemic preparedness and intersectoral collaborations. A contextual intersectoral collaboration framework and/or streategies should be formalized through different public sectors along with the academia, research, NGOs and other leading institutions.

To sum up, this study offers novel insights into the operational realities of CHWs in a densely populated urban Indian context during the COVID-19 crisis. The documentation of intersectoral collaboration models, informal task shifting, and gaps in system preparedness provides valuable direction for designing resilient urban healthcare systems for future pandemics.

## Supplementary Information

Supplementary Material 1.

## Abbreviations

AMC: Ahmedabad Municipal Corporation
ANW: Auxiliary Nurse Midwife
ASHA: Accredited Social Health Activist
AWW: Anganwadi Worker
BRTS: Bus Rapid Transit System
CHC: Community Health Center
CHW: Community Health Worker
COVID-19: Coronavirus Disease 2019
FHW: Female Health Worker
HIV: Human Immunodeficiency Virus
IDI: Infectious Disease (Context dependent, could also be Infectious Diseases Institute or Informatics)
IFA: Iron Folic Acid
IRS: Indoor Residual Spraying
NCD: Non-Communicable Disease
NGO: Non-Governmental Organization
OPD: Outpatient Department
PNC: Postnatal Care
PPE: Personal Protective Equipment
RMNCH + A: Reproductive, Maternal, Newborn, Child Health + Adolescent
SMC: Supplementary Measles Campaign (Context dependent, could also be Seasonal Malaria Chemoprevention)
TB: Tuberculosis
THR: Take-Home Ration
VHND: Village Health and Nutrition Day
