# Supplementary material for: Challenges in delivering urban healthcare services during COVID-19 pandemic: a mixed-methods study in Ahmedabad, India
Source: BMC Health Serv Res. 2025 Jul 26;25:979. doi: 10.1186/s12913-025-13147-0 (PMC12297839; doi:10.1186/s12913-025-13147-0)
Supplement: Supplementary file 1 — Supplementary Material 1. [file 12913_2025_13147_MOESM1_ESM.pdf]

Form 2 [Community Health workers]: Routine and/or essential service delivery during the COVID19 pandemic in Ahmedabad

(Administer this questionnaire to the Community Health workers in Ahmedabad)

Routine services: *Routine services are those services, which are routinely (day to day basis) carried out in health care facility without any adverse event or disturbance of Health system.*

Essential Services: *Essential services are those routine services, which are prioritized when there is some adverse event or resilience of Health system or any kind of disturbance in the health system.*

Section-1 (Basic Details)

UID		Date	
Name of the hospital			
Name of the respondent			
Designation	0. ASHA<br>1. AWW<br>2. MPHW<br>3. ANM<br>4. Other (specify)______	Gender	0. Female<br>1. Male

Section-2 (In-depth Interview guide)

1. Can you please brief us about the functionality of your health care facility during the pandemic? (Probe: Routine activities vs pandemic activities – the differences-
   - Routine work (day to day activities) / specialty related duties– explain in detail
   - Any disruption in the planned follow up treatment or surgeries
2. How have you ensured routine services during the pandemic? (Probe: During the lockdown, 1st wave, and the subsequent waves)
3. How have you ensured the essential services during the pandemic? (Probe: Immunization, Delivery care, ANC/PNC check-ups etc.)
4. How have you ensured the emergency services during the pandemic? (Probe: Which emergency services were provided to the patients and any challenges? Scheduled surgeries, Ambulance services etc.)
5. How was the health workforce managed during the pandemic?
   - Probe: Additional HRs (medical students, interns, Paramedical doctors), Support system from govt., Coordination activities etc.
   - Probe: Task shifting of staff/ Work distribution among the existing, new HRs
     -Ward boys/nurses, / medical interns were involved in RTPR testing.
     -Paramedical/public health interns were involved in the management of immunization services
     -How it has changed with increasing complications, shortage of beds, oxygen, or materials & overload of activities)
6. Was there any integration of technology in providing service delivery? (For which services: Routine/essential, emergency or Just COVID related) (Probe: Use of Mobile Van, Telemedicine)
7. Please brief us about the information flow and communications from the higher authority.
8. Please tell us about the capacity-building activities for pandemic management. (Probe: How were you trained to manage the situation, any special e-Training, etc.
9. Are there any other departments you collaborated with apart from health sector for pandemic management at your facility? or Who are the stakeholders associated with you apart from your healthcare facility or department for management of the pandemic?

10. Can you please tell us about your role and responsibilities during COVID 19 pandemic management?
11. Can you brief us about the routine clinical task done by you?

    (Probe: Was there any change in tasks or any additive roles during the COVID19 pandemic? Any task shifting? Repurposing of Health care workers to support COVID-19 services?, Prioritizing tasks)
12. Can you tell us about your motivation for working during a pandemic? How you feel and coped with these difficult times?

    (Probe: motivated to hard work, anxiety, overload of work)
13. Have you ever been in epidemic or outbreak management task force earlier and have you collaborated with other sectors apart from health sectors?

Section-3 (Intersect oral collaboration details)

Who is your point of contact during the COVID19 management (show them the COVID19 task force list) within the health system and outside the health system or vice-versa? Rate them based on the working relation (T: Type) and how frequently you are collaboratively working in different scenarios (F: Frequency)?

**Type/ Frequency**	**During the 1st wave**	**Post 1st wave**	**During the 2nd wave**	**Post 2nd wave**	**During the 3rd wave**	**Post 3rd wave**
**Actor-1:**						
T						
F						
**Actor-2:**						
T						
F						
**Actor-3:**						
T						
F						
**Actor-4:**						
T						
F						
**Actor-5:**						
T						
F						
**Actor-6:**						
T						
F						
**Type:** 1. Not linked (Do not work together), 2. Communication (share information only), 3. Cooperation (Work together informally to achieve common goals), 4. Collaboration (Work together as a formal team with specific responsibilities), 5. Fully linked (Work together as a formal team, mutually plan & share staff or resources to accomplish goals)<br>**Frequency:** 1. No contact 2. Daily, 3. Twice weekly 4. Once Weekly, 5. Twice Monthly, 6. Once Monthly						

Hints: Education department, Media/Journalists, Transportation department, Police department, Veterinary cell (Government & Private Veterinarians), Livestock inspectors/Animal workers, NGOs, Research institutes.
